# Metabolomics Pilot Study Identifies Desynchronization of 24-H Rhythms and Distinct Intra-patient Variability Patterns in Critical Illness: A Preliminary Report

**DOI:** 10.3389/fneur.2020.533915

**Published:** 2020-10-02

**Authors:** Elizabeth R. Lusczek, Lee S. Parsons, Jesse Elder, Stephen B. Harvey, Mariya Skube, Sydne Muratore, Greg Beilman, Germaine Cornelissen-Guillaume

**Affiliations:** ^1^Department of Surgery, University of Minnesota, Minneapolis, MN, United States; ^2^Minnesota Supercomputing Institute, University of Minnesota, Minneapolis, MN, United States; ^3^Bioinformatics and Computational Biology, University of Minnesota, Minneapolis, MN, United States; ^4^Center for Mass Spectrometry and Proteomics, University of Minnesota, Minneapolis, MN, United States; ^5^Department of Integrative Biology and Physiology, University of Minnesota, Minneapolis, MN, United States

**Keywords:** circadian rhythms, metabolomics, critical illness, ICU—intensive care unit, cortisol (Cor), precision medicine

## Abstract

**Background:** Synchronized circadian rhythms play a key role in coordinating physiologic health. Desynchronized circadian rhythms may predispose individuals to disease or be indicative of underlying disease. Intensive care unit (ICU) patients likely experience desynchronized circadian rhythms due to disruptive environmental conditions in the ICU and underlying pathophysiology. This observational pilot study was undertaken to determine if 24-h rhythms are altered in ICU patients relative to healthy controls by profiling 24-h rhythms in vital signs and plasma metabolites.

**Methods:** We monitored daily rhythms in 5 healthy controls and 5 ICU patients for 24 h. Heart rate and blood pressure were measured every 30 min, temperature was measured every hour, and blood was sampled for mass spectrometry-based plasma metabolomics every 4 h. Bedside sound levels were measured every minute. Twenty-four hours rhythms were evaluated in vitals and putatively identified plasma metabolites individually and in each group using the cosinor method.

**Results:** ICU patient rooms were significantly louder than healthy controls' rooms and average noise levels were above EPA recommendations. Healthy controls generally had significant 24-h rhythms individually and as a group. While a few ICU patients had significant 24-h rhythms in isolated variables, no significant rhythms were identified in ICU patients as a group, except in cortisol. This indicates a lack of coherence in phases and amplitudes among ICU patients. Finally, principal component analysis of metabolic profiles showed surprising patterns in plasma sample clustering. Each ICU patient's samples were clearly discernable in individual clusters, separate from a single cluster of healthy controls.

**Conclusions:** In this pilot study, ICU patients' 24-h rhythms show significant desynchronization compared to healthy controls. Clustering of plasma metabolic profiles suggests that metabolomics could be used to track individual patients' clinical courses longitudinally. Our results show global disordering of metabolism and the circadian system in ICU patients which should be characterized further in order to determine implications for patient care.

## Introduction

Circadian rhythms span multiple levels of hierarchical organization in biological systems, through molecular processes (peripheral oscillators) to whole-body rhythms such as core body temperature (1). Synchronized, intact rhythms are fundamental to human health and their desynchronization is now known to contribute to morbidity and pathophysiology in otherwise healthy individuals (2–4). Critical care physicians increasingly recognize the influence of circadian desynchronization on physiology and outcomes in critically ill patients (5, 6). There is clear evidence that supporting circadian rhythms in cancer patients contributes to higher quality of life (7). Likewise, it may be beneficial to support circadian rhythms in intensive care unit (ICU) patients (8, 9).

While there is evidence that circadian rhythms are disrupted in the critically ill, the etiology is complex and multifaceted (10). A host of desynchronizing influences are present in critical illness, including poor sleep (11), continuously-administered parenteral nutrition (12–14), unregulated light/dark cycles and sound levels (15–17), as well as the influence of pharmacological treatments (18).

Due to the hierarchical and widely distributed organization of the circadian system, multiple data types should be collected to determine the extent of circadian desynchronization in critical illness regardless of the underlying cause. High-throughput—omics data, particularly metabolomics, is suited to this endeavor, since metabolism is tightly coupled to the core clock mechanism (19–21). Multiple circadian metabolites have been reported in the literature, identified with mass spectrometry-based metabolomics (22–24). Alterations to circadian rhythms in the metabolome have been documented in shift work (25) and sleep disruption protocols (26).

We collected preliminary data to evaluate 24-h rhythms in ICU patients in multiple data types: heart rate, blood pressure, temperature, and mass spectrometry-based metabolomics in this observational study. We also measured sound levels in participants' rooms. We hypothesized that disruptions to 24-h rhythms in ICU patients relative to healthy controls would be identified in multiple metabolites and vital signs. To test this hypothesis, we conducted a pilot study in 5 healthy controls and 5 ICU patients. This study is the first to use metabolomics to profile circadian rhythms in the critically ill.

## Methods

The study protocol (study number 1505M70361) was reviewed and approved by the University of Minnesota Institutional Review Board in accordance with the Code of Federal Regulations, 45 CFR 46.101(b). All enrolled healthy participants gave their informed consent to study staff. Informed consent was obtained from legally authorized representatives of all enrolled ICU patients since they were sedated and unable to consent.

### Patient Population

From 2015 to 2016, we collected data in 5 healthy control participants and 5 intensive care unit (ICU) patients for 24 h. Healthy participants were over 18 years of age with a self-reported average of 7–8 h of sleep a night, and were in generally good health. They were screened to exclude the following factors that may affect normal circadian rhythms: shift work, blindness, use of over-the-counter sleep aids or supplements, history of traumatic brain injury, brain surgery, or sleep apnea, smoking, use of psychotropic drugs, or current alcohol use of more than 2 drinks per day. Healthy participants were monitored by trained staff at the University of Minnesota's Masonic Clinical Research Unit. Catheters were placed peripherally to facilitate blood draws. They were not subjected to constant routine conditions. They stayed in single rooms with windows allowing natural light. They were provided meals from a cafeteria at times of their choosing that generally corresponded to set mealtimes, controlled the room's light levels to their preference, and determined their own periods of rest and activity. All 5 participants reported that they slept well despite nighttime monitoring.

ICU patients were recruited from the University of Minnesota Medical Center ICU according to the following criteria: age >18 years, mechanical ventilation with sedation for at least 3 days, and any of the following: emergent surgery, emergent intubation, admission after severe trauma, damage control surgery, or admission to ICU for post-operative complication. Patients with these conditions were excluded: severe nervous system disorder such as traumatic brain injury or recent neurosurgery, confirmed or suspected drug overdose or alcohol abuse, blindness, severe renal failure, end stage liver disease or hepatic encephalopathy, extracorporeal membrane oxygenation (ECMO) use, required vasopressors or steroids, central sleep apnea, hemoglobin levels ≤8 g/dL, and dementia or delirium prior to ICU stay. All ICU patients were fed enterally.

Twelve participants were enrolled in the study. One healthy control was disqualified because of abnormally high blood pressure. One ICU patient extubated during the study and was disqualified.

### Data Collection

In all research participants, beginning at 9:00 a.m., heart rate and blood pressure were measured every 30 min. Temperature was measured every hour orally (healthy controls) or *via* Foley catheter (ICU patients). Bedside sound levels were measured every minute (Bruel and Kjaer 2250L, Duluth, GA).

For the metabolomics portion of the study, blood samples (5 mL) were drawn for metabolomics analysis every 4 h beginning at 9:00 a.m. in each participant. Plasma was extracted and aliquots were stored at −80°C until preparation for analysis with mass spectrometry. Reagents were obtained from Fisher Chemical Co., and were of LC-MS grade or better. Samples were prepared according to published protocols (27, 28). Internal standards (see Supplementary Table 1) were added to thawed plasma samples followed by the addition of 4 volumes of cold (−20°C) 10% acetone 90% methanol (i.e., added 400 μL solvent to 100 μl sample). Samples were vortexed and incubated at −20°C for 15 min. Samples were spun down at 13,000 g for 10 min at 4°C and the supernatant transferred to a clean tube. The incubation and centrifugation were repeated and the final supernatant transferred to a clean tube, which was then dried under nitrogen. Preparation for reverse-phase LC-MS analysis was completed by adding a starting buffer (100 μl of 5% acetonitrile, 95% water, 0.1% formic acid). Sample pH was adjusted to ~2 by adding 10–20 μl of 10% formic acid.

Samples were analyzed *via* chromatographic separation in-line with mass spectrometry. Ultra-high performance liquid chromatography (UHPLC) was performed using the Thermo Scientific Ultimate 3000 UHPLC platform. For reverse-phase analysis the instrument was fitted with a Waters Acquity BEH C-18 column (2.1 × 100 mm, 1.7 μm particle size). Flow rate was 0.4 mL/min and the column compartment was set to 40°C. Ten microliters were injected onto the column. Elution solutions were (A) water with 0.1% formic acid and (B) acetonitrile with 0.1% formic acid. The elution gradient was as follows: 2% B for 0.5 min; increase to 25% B over the next 0.5 min; increase to 80% B over the next 7 min; increase to 100% B for 2 min; decrease to 2% B over the next 0.5 min; hold at 2% B for 2.5 min.

The Q Exactive™ Quadrupole-Orbitrap mass spectrometer (Thermo Fisher Scientific, Waltham, MA) was employed for mass analysis. The instrument profiles for both high mass accuracy (<2 ppm) and spectral resolution (typically at 70,000 resolution in full scan mode). Analysis was performed in positive mode over a mass range of 70–1,050 m/z.

### Data Analysis

#### Evaluation of Sound Levels

Differences in bedside A-weighted equivalent continuous sound levels in participants' rooms were evaluated using Wilcoxon Rank Sum tests with the R software package v.3.3.3 (29). Sound meter data were discarded for three study subjects (one healthy control and two ICU patients) due to technical failure. There was very little variation in the sound levels recorded for these three participants (mean LAeq = 49.0 dB, standard deviation = 7.03 dB for analyzed data; mean LAeq = 25.7 dB, standard deviation = 0.184 dB for discarded data).

#### Evaluation of 24-H Rhythms

Time series measurements of each participant's heart rate, blood pressure, temperature, and plasma metabolite intensities were evaluated for 24-h rhythmicity using the cosinor methodology (30) in individuals and at the population level. MESOR (midline estimating statistic of rhythm or rhythm-adjusted mean), amplitude, and acrophase were determined for each variable in each individual.

Population means for each variable were evaluated and compared between the two study groups using parameter tests for the population mean cosinor (PMC) (30). Inference is done by (1) calculating the arithmetic mean of individual MESORs and (2) calculating the average of individual amplitude/acrophase pairs, treating the pairs as vectors. PMC *p*-values indicate the statistical significance of the zero-amplitude test and reflect similar amplitude-acrophase pairs among individuals in the population at the trial period considered (i.e., 24 h), irrespective of whether a 24-h rhythm could be demonstrated individually. Note that PMC can show a significant result when the combined *p*-values do not because the PMC algorithm uses all the data at once, increasing the statistical power over the individual calculations.

#### Mass Spectrometry Metabolomics

Plasma metabolites were putatively identified with the Progenesis QI (Non-linear Dynamics, Durham, NC) and Xcalibur (Thermo Fisher Scientific, Waltham, MA) software packages in conjunction with the Metlin (31) and HMDB (32) databases and confirmed using an in-house database compiled by the facility performing the spectrometry. A group of 60 previously identified circadian metabolites were targeted (24, 33). Analysis of 24-h rhythms was limited to these 60 putatively identified metabolites.

Principal component analysis of all spectral features identified by Progenesis QI was performed using the R software package after intensities were log-transformed (29). Features were filtered using the ANOVA algorithm in Progenesis (*q* < 0.05) when comparing ICU patients to healthy controls (34). Filtering resulted in a list of 15,000 identified features distinguishing ICU patients from healthy controls. This reduced dataset was linked with HMDB identifiers by Progenesis QI and exported directly to Ingenuity Pathway Analysis (QIAGEN Inc., https://www.qiagenbioinformatics.com/products/ingenuity-pathway-analysis/). IPA was able to map 12,825 of these HMDB IDs to its database. For features that match more than one HMDB identity, IPA resolves duplicates by choosing the feature with the lowest *p*-value. Following the resolution of duplicates, the resulting dataset of 2,696 metabolites with HMDB IDs was used by IPA in the pathway analysis.

Serum cortisol, quantified by Fairview Hospital Laboratories, was used to confirm cortisol quantified by mass spectrometry.

## Results

Healthy controls (two males and three females) were 45–72 years of age. ICU patients (two males and three females) were 43–66 years of age. Patient characteristics are described in Table 1. BMI data for both groups is shown in Supplementary Table 2, and did not differ between the groups.

**Table 1:** Pilot study ICU patient characteristics.

**Patient ID**	**APACHE II**	**Discharge destination**	**Primary diagnosis**	**Length of stay**
P07	6	Home	Tracheal stenosis	8 days
P08	11	Home	Tricuspid valve regurgitation	30 days
P10	9	Long-Term Acute Care	Necrotizing pneumonia, bronchopleural fistula	23 days
P11	23	Long-Term Acute Care	ARDS of unknown origin	28 days
P12	14	Long-Term Acute Care	Abscess requiring emergent tracheostomy	23 days

*ICU patient demographics, APACHE II scores, and outcomes. ARDS, Acute Respiratory Distress Syndrome; APACHE, Acute Physiology and Chronic Health Evaluation*.

**Figure 1:**
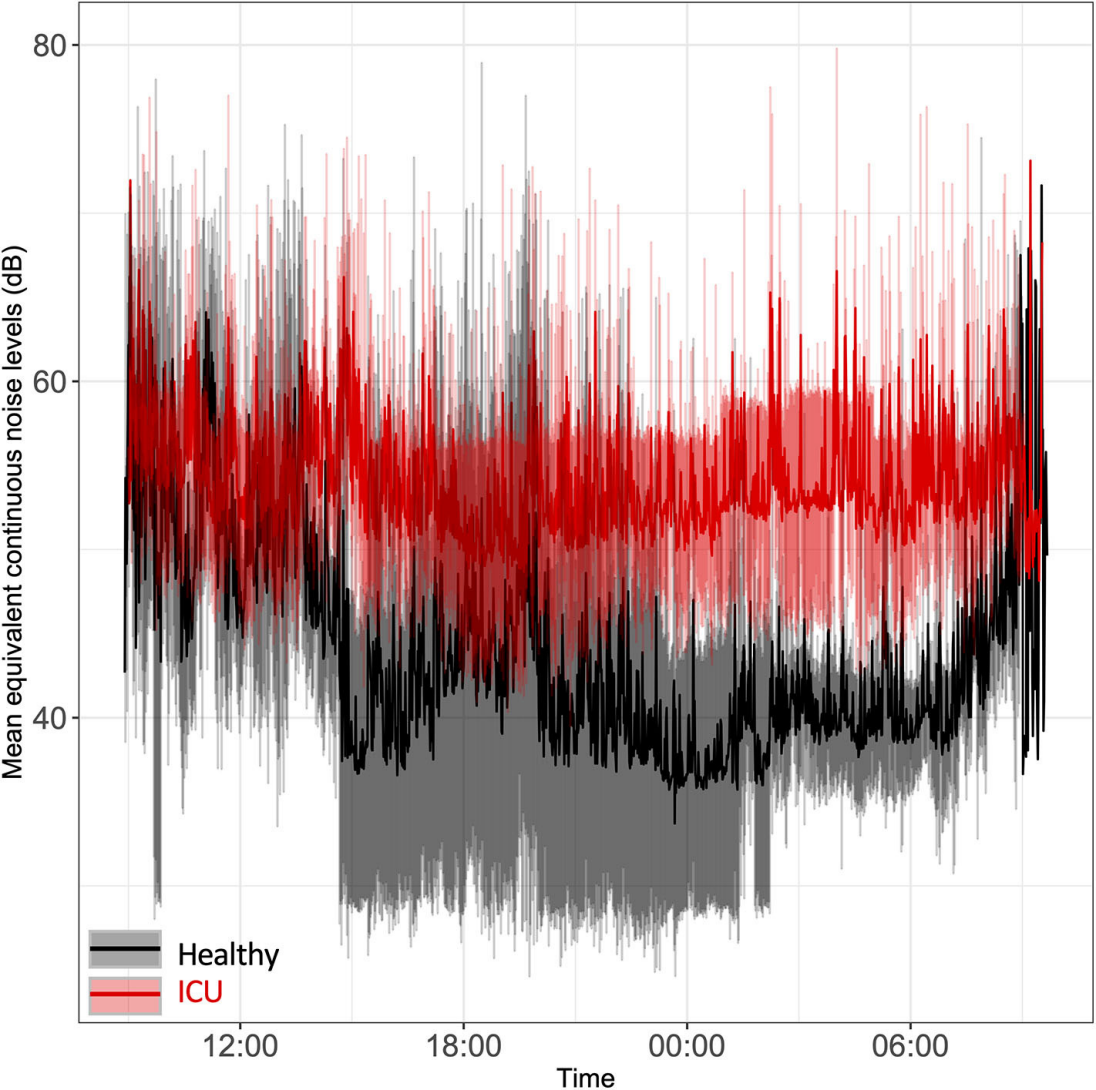
Time series plot of the mean equivalent continuous noise levels. Bedside noise levels were recorded in the rooms of ICU patients (red line) and healthy controls (black line). Light red shading represents the standard deviation for ICU patients; gray shading represents the standard deviation for healthy controls.

Sound levels were significantly higher in ICU patients' rooms than in healthy controls' rooms, particularly at night (Figure 1). Average minimum equivalent continuous noise levels (LAeq) were 48 dB (ICU) vs. 33 dB (controls; *p* < 0.0001). Sound levels in ICU patient rooms were, on average, above recommended World Health Organization limits of 35 dB (35). In two of the patients' rooms, the minimum sound levels were quite loud: 43.5 and 54.9 dB, respectively. Mean noise levels overlapped in ICU patient rooms and healthy controls' rooms from 9:00 to 13:30 and again at 9:00 the following morning. Noise levels remained elevated in ICU patients' rooms even during nighttime hours while healthy controls had a clear decrease in sound levels at night, particularly around midnight. We noted a clear lack of a 24-h pattern in ICU noise levels relative to healthy controls.

### 24-H Rhythms in Vitals

Twenty-four hours rhythms exist both individually and at the group level in heart rate and blood pressure for healthy controls (Table 2). Isolated individual ICU patients showed significant rhythms in vital signs. The presence of circadian rhythms in vitals at both the individual and group levels in healthy controls shows that these individuals had similar amplitudes and acrophases.

**Table 2:** Vitals and putatively identified metabolites with significant 24-h rhythms.

	**Number of participants with significant rhythmicity (*p* < 0.05)**	**PMC *p*-value**
**Heart rate**		
HEA	5	0.003
ICU	2	0.433
**Systolic blood pressure**		
HEA	4	0.069
ICU	2	0.679
**Diastolic blood pressure**		
HEA	4	0.007
ICU	2	0.651
**Temperature**		
HEA	1	0.091
ICU	1	0.778
**Cortisol (mass spectrometry)**		
HEA	1	0.033
ICU	1	0.166
**Cortisol (laboratory)**		
HEA	2	0.009
ICU	1	0.035
**Cortisone**		
HEA	2	0.020
ICU	0	0.251
**Lyso PC 18.2**		
HEA	1	0.016
ICU	0	0.259
**Lyso PE 18.1**		
HEA	3	0.037
ICU	0	0.480
**Lyso PE 18.2 (a)**		
HEA	1	0.039
ICU	0	0.269
**Lyso PE 18.2 (b)**		
HEA	2	0.043
ICU	0	0.636
**Proline**		
HEA	2	0.039
ICU	2	0.622
**Tyramine**		
HEA	1	0.021
ICU	1	0.252
**Acetylcarnitine**		
HEA	0	0.012
ICU	1	0.065
**Aminoadipate**		
HEA	0	0.030
ICU	0	0.504
**2-Hydroxylauroylcarnitine**		
HEA	0	0.008
ICU	1	0.393

*The number of participants exhibiting significant 24-h rhythmicity for each variable and population mean cosinor (PMC) p-values for variables monitored in this study. A total of 29 rhythmic features were observed in healthy controls, while only 14 were observed in ICU patients(p = 0.013). HEA, Healthy Controls; ICU, ICU patients*.

**Figure 2:**
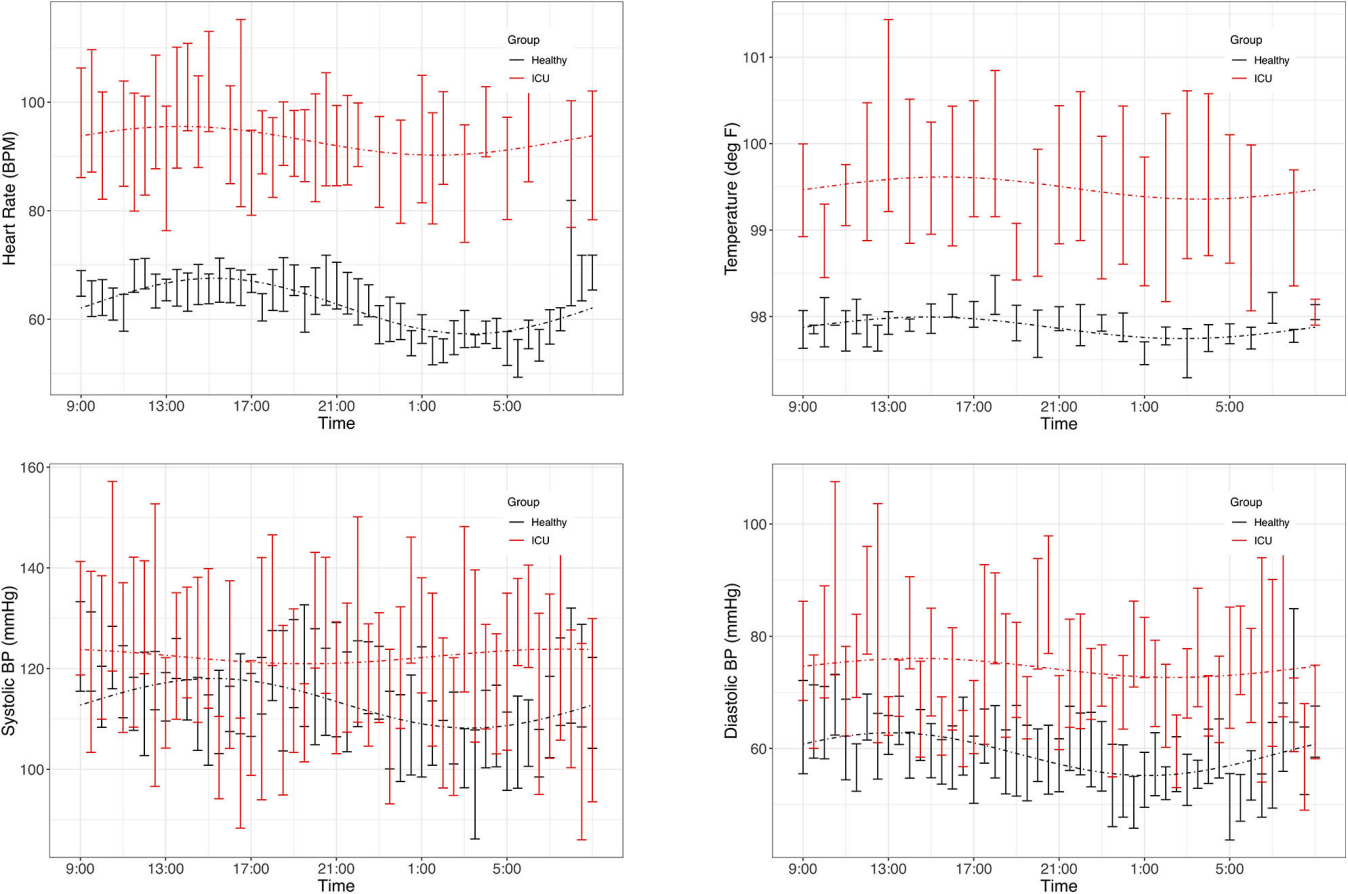
Time series plots of vitals. Data are presented as mean and standard error in ICU patients (red) and healthy controls (black). Clockwise: heart rate, temperature, diastolic blood pressure, systolic blood pressure. Dashed lines represent the PMC fits of the 24-h rhythm to the data. Plots of the individual data are presented in Supplementary Data Sheet 1.

As a group, healthy controls had significant (PMC *p* < 0.007) 24-h rhythms in heart rate and diastolic blood pressure, and near-significant (PMC *p* < 0.091) rhythms in temperature and systolic blood pressure (Figure 2 and Table 2). As a group, ICU patients had no significant 24-h rhythms in the same variables, even though some individual patients had a significant rhythm. This indicates a lack of coherence in the acrophases and amplitudes of ICU patients in these variables. This can also be seen in Table 3, which reports 24-h rhythm characteristics (24-h *p*-value, MESOR, amplitude, and acrophase) determined by PMC. For ICU patients, 95% confidence limits could not be computed for the majority of the variables measured.

**Table 3:** PMC rhythm characteristics in vitals and putatively identified metabolites with significant 24-h rhythms.

**Variable**	**Group**	**PMC *p*-value**	**MESOR (95% CI)**	**Amplitude (95% CI)**	**Acrophase**	**Putative metabolite**
					**(95% CI)**	**identity**
Heart rate	Healthy	*p* = 0.003	62.4 (55.4, 69.4)	5.13 (4.08, 6.18)	15:16 (12:53, 17:56)	
	ICU	*p* = 0.433	92.9 (69.4, 116)	2.65 (NS)	13:40 (NS)	
Systolic blood pressure	Healthy	*p* = 0.069	113 (94.5, 132)	4.93 (NS)	15:16 (NS)	
	ICU	*p* = 0.679	122 (89, 155)	1.46 (NS)	7:36 (NS)	
Diastolic blood pressure	Healthy	*p* = 0.007	59 (46.4, 71.6)	3.81 (2.65, 4.97)	13:10 (9:40, 17:08)	
	ICU	*p* = 0.651	74.4 (54.7, 94.1)	1.71 (NS)	14:24 (NS)	
Temperature	Healthy	*p* = 0.091	97.9 (97.7, 98.1)	0.125 (NS)	14:48 (NS)	
	ICU	*p* = 0.778	99.5 (97.5, 102)	0.128 (NS)	15:36 (NS)	
Cortisol (lab)	Healthy	*p* = 0.009	6.9 (5.85, 7.95)	4.82 (2.92, 6.72)	9:24 (8:36, 10:44)	
	ICU	*p* = 0.035	20.5 (8.7, 32.3)	2.84 (1.45, 4.23)	10:08 (6:04, 13:52)	
MS feature RT = 3.38, m/z = 363.2160	Healthy	*p* = 0.033	33.75 (31.57, 35.93)	16.96 (8.585, 25.33)	8:44 (6:16, 11:52)	Cortisol
	ICU	*p* = 0.116	72.90 (42.72, 103.1)	13.99 (NS)	11:08 (NS)	
MS feature RT = 5.65, m/z = 342.2633	Healthy	*p* = 0.008	85.1 (59.3, 111)	27.3 (14, 40.6)	10:16 (8:36, 13:32)	2-hydryoxylauroylcarnitine
	ICU	*p* = 0.393	198 (82, 314)	42.1 (NS)	12:56 (NS)	
MS feature RT = 0.83, m/z = 186.1123	Healthy	*p* = 0.012	180 (104, 257)	37.1 (14.3,59.9)	19:20 (17:52, 22:52)	Acetylcarnitine
	ICU	*p* = 0.065	232 (55, 409)	52.9 (NS)	2:24 (NS)	
MS feature RT = 0.97, m/z = 162.0759	Healthy	*p* = 0.030	82.9 (71, 94.8)	6.47 (1.09, 11.9)	2:32 (00:04, 7:52)	Aminoadipate
	ICU	*p* = 0.504	154 (84.5, 224)	5.72 (NS)	8:32 (NS)	
MS feature RT = 3.41, m/z = 361.2011	Healthy	*p* = 0.020	477 (393, 561)	171 (103, 239)	9:36 (6:32, 12:56)	Cortisone
	ICU	*p* = 0.251	548 (350, 746)	47.8 (NS)	10:32 (NS)	
MS feature RT = 8.00, mass = 519.3318 (neutral mass)	Healthy	*p* = 0.016	223 (191, 255)	30.8 (18.4, 43.2)	19:24 (15:32, 22:32)	LysoPC 18.2
	ICU	*p* = 0.259	130 (92.4, 168)	10.6 (NS)	14:00 (NS)	
MS feature RT = 8.58, mass = 479.3002 (neutral mass)	Healthy	*p* = 0.037	424 (258, 590)	147 (74.8, 219)	19:24 (17:32, 21:20)	LysoPE 18.1
	ICU	*p* = 0.480	249 (111, 387)	35.4 (NS)	00:40 (NS)	
MS feature RT = 7.95, mass = 477.2845 (neutral mass)	Healthy	*p* = 0.039	83.9 (59.2, 109)	24.3 (8.1, 40.5)	19:12 (15:12, 21:12)	LysoPE 18.2 (a)
	ICU	*p* = 0.269	32.9 (21.8, 44)	3.5 (NS)	00:44 (NS)	
MS feature RT = 7.76, m/z = 478.2921	Healthy	*p* = 0.043	120 (98.7, 141)	16.7 (7.22, 26.2)	18:04 (15:24, 21:48)	LysoPE 18.2 (b)
	ICU	*p* = 0.636	42.3 (21.6, 63)	2.42 (NS)	20:00 (NS)	
MS feature RT = 0.67, m/z = 116.0707	Healthy	*p* = 0.039	105 (69.2, 141)	19.9 (6.3, 33.5)	21:00 (18:04, 22:08)	Proline
	ICU	*p* = 0.622	119 (69.5, 169)	5.5 (NS)	1:16 (NS)	
MS feature RT = 1.93, m/z = 138.0913	Healthy	*p* = 0.021	87.1 (54.2, 120)	19.5 (9.52, 29.5)	6:36 (3:12, 11:08)	Tyramine
	ICU	*p* = 0.112	105 (35.1, 175)	31.3 (NS)	10:40 (NS)	

*Twenty-four hours rhythm characteristics of vital signs and putatively identified metabolites quantified with mass spectrometry, quantified with PMC. MESOR is the midline estimating statistic of rhythm, amplitude is the circadian amplitude, and acrophase reflects the timing of overall high values recurring each day, expressed in clock time. Confidence intervals for insignificant 24-h rhythms are reported as NS (not significant). Mass spectrometry features are uniquely identified by retention time (RT) and mass to charge ratio (m/z) or neutral mass. Putative metabolite identities were established using published literature and online databases (METLIN, HMDB), and were confirmed with in-house databases at the University of Minnesota's Center for Mass Spectrometry and Proteomics*.

### 24-H Rhythms in the Metabolome

Progenesis QI identified 25,000 unique spectral features. Sixty metabolites were putatively identified using retention times and mass-to-charge ratios published in the literature (24, 33), online databases (METLIN, HMDB) and verified with in-house lists curated by the Center for Mass Spectrometry and Proteomics. We ran both individual cosinor and PMC on these 60 putatively identified metabolites. Of these 60 metabolites, PMC showed that 10 had a significant 24-h rhythm in the healthy control group (Table 3 and Supplementary Data Sheets 1, 2). None had a significant rhythm in the ICU group. For brevity, we present information only on the 10 significant metabolites. We note that there was some ambiguity about the identification of Lyso PE 18.2, as two features with similar intensities, mass-to-charge ratios, and retention times were identified in the dataset. Both features are presented as Lyso PE 18.2 (a) and Lyso PE 18.2 (b).

**Figure 3:**
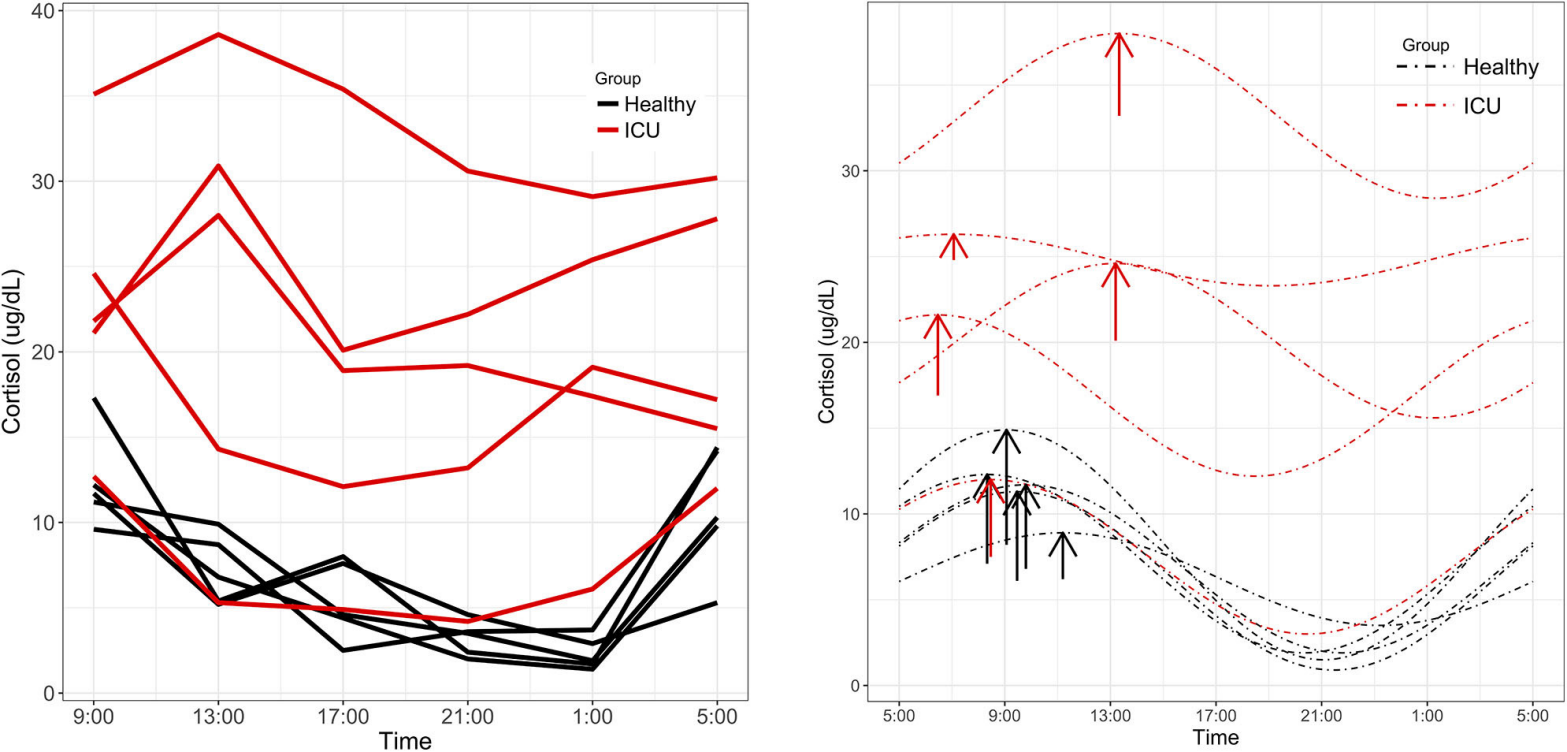
Cortisol rhythms. **(Left)** Individual time series plots of cortisol concentrations quantified by Fairview Hospital Laboratories. Red lines indicate measured cortisol concentrations in ICU patients and black lines indicate healthy controls. **(Right)** ICU patients showed alterations in MESOR and phase not present in controls. Dashed lines represent cosinor fits to the data. Arrows are placed at each rhythm's acrophase. Arrow lengths reflect rhythm amplitude.

Significant 24-h rhythms were identified in cortisol both individually and at the group level in healthy controls and in ICU patients (Laboratory value, Table 2). Even though PMC showed that both the ICU group and the healthy control group had a significant 24-h rhythm in cortisol, patients' cortisol rhythms had some abnormal features: they had a higher mean (20.5 vs. 6.9 μg/dL, *p* = 0.01) than controls as well as scattered acrophases (Figure 3). Healthy controls had tightly clustered acrophases [9:24 (8:36, 10:44)] while ICU patients did not [10:08 (6:04, 13:52); data are reported as acrophase (95% confidence interval)]. The scatter of phases in the ICU patient group leads to a significant lowering of rhythm amplitude in the ICU patient group relative to the healthy control group as quantified by PMC (cortisol was quantified by the hospital laboratory and with mass spectrometry, showing good agreement in patterns (see Supplementary Figure 1)].

For the 10 putatively identified rhythmic metabolites, some patterns were evident in the data. For cortisone, Lyso PC 18.2, Lyso PE 18.1, Lyso PE 18.2, and proline, rhythms exist both at the individual level and the group level in healthy controls. Rhythms do not exist in ICU patients at either among individuals or as a group, except proline has a rhythm at the individual level in ICU patients.

For acetylcarnitine, aminoadipate, tyramine, and 2-hydroxylauroylcarnitine, neither patients nor controls have significant rhythms when evaluated individually. We interpret this to mean that patients did not differ much from healthy controls individually in these variables. However, PMC showed the healthy control group had significant rhythms while ICU patient group did not. This implies that among ICU patients, there was more variability among individuals in amplitude, MESOR, and acrophase. Among healthy controls, however, there was much less variability among individuals in 24-h rhythm characteristics.

### Principal Component Analysis

**Figure 4:**
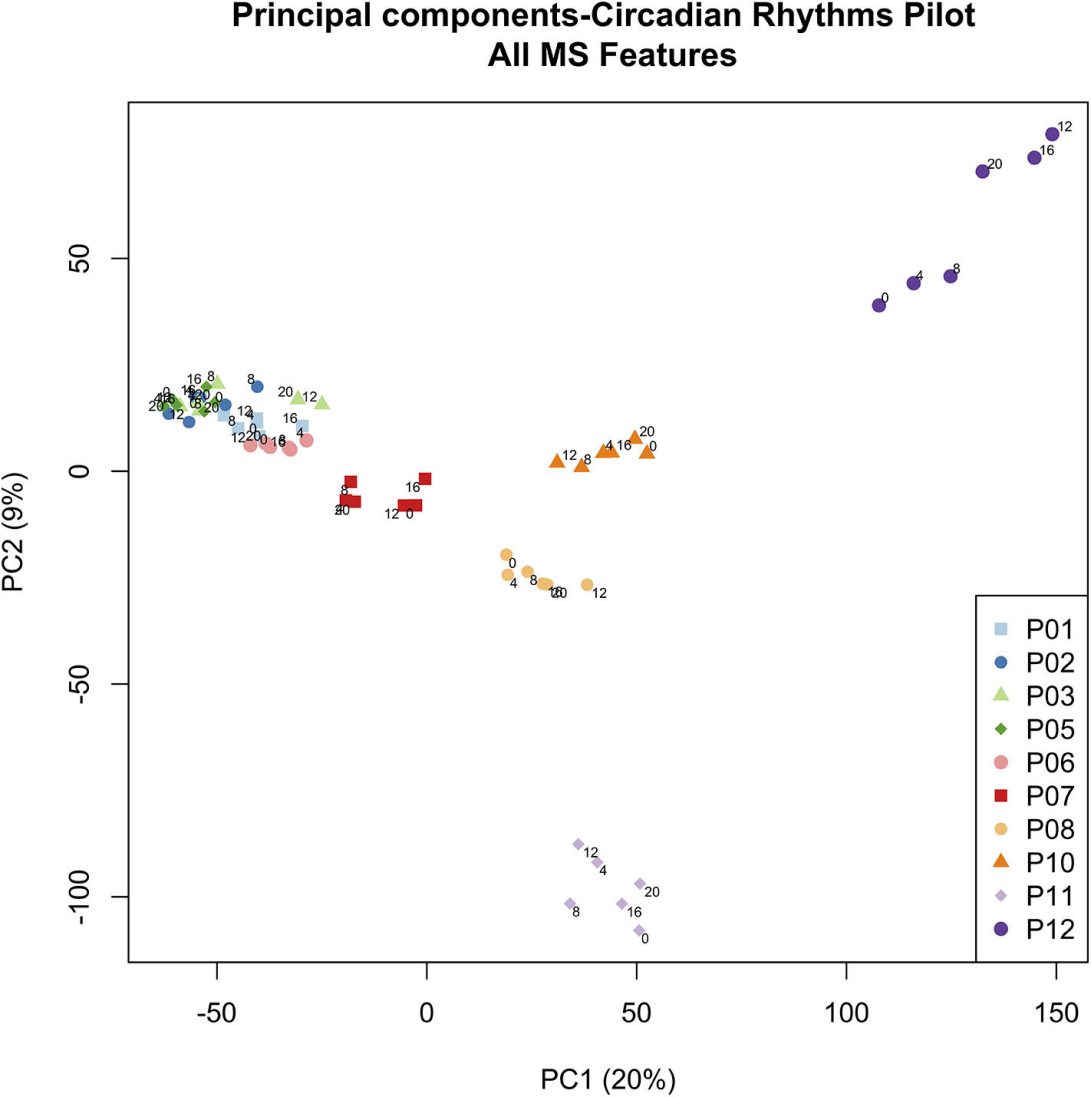
PCA scores plot of metabolic profiles. Scores from the first two principal components were constructed from a total of 60 plasma samples. Samples were obtained from 5 healthy controls (P01–P06, clustered on the left hand side) and 5 ICU patients (P07–P12). Each colored dot corresponds to an individual plasma sample. Each ICU patient is clearly visible in the PCA scores (dark red, orange, yellow, light purple, dark purple). Small numbers indicate the hour at which the plasma sample was obtained (0, 4, 8, 12, 16, 20 h). Zero hour corresponds to 9:00 a.m. for all participants. Figure 4 © 2019 Lusczek ER. Published in Metabolomics—New Insights into Biology and Medicine under CC BY 3.0 license. Available from (36).

To more globally assess differences in metabolism between healthy controls and ICU patients, we performed principal component analysis (PCA) on the mass spectrometry data. A scores plot (Figure 4) of the features identified by Progenesis QI shows that each ICU patient's plasma samples (P07–P12) are strongly clustered together away from healthy controls (P01–P06). Of the 5 ICU patients studied, 4 had ICU stays of >20 days and 3 were discharged to long-term acute care facilities (Table 1). The 5th patient had an 8-day stay and was discharged home. This patient's cortisol rhythm was closest to the rhythms of healthy controls (Figure 3) and their metabolic profiles (red circles) closest to the cluster of healthy controls (Figure 4). A version of Figure 4 appears in the book Metabolomics—New Insights into Biology and Medicine (36).

To reduce this dataset, features were evaluated for statistical significance in Progenesis QI by comparing feature intensity in the ICU group vs. feature intensity in the healthy control group. Features with ANOVA *q* < 0.05 were retained. This reduced the data set from 25,000 identified features to 15,000 features. Since this is still a very large dataset, we explored it with Ingenuity Pathway Analysis (IPA). The canonical pathway analysis showed 17 pathways dysregulated in ICU patients relative to healthy controls at the level of *p* < 0.05 (Supplementary Table 3). The top 5 dysregulated pathways were eicosanoid signaling, prostanoid biosynthesis, FXR/RXR activation, spermine biosynthesis, and spermidine biosynthesis I. A striking 68 different diseases and functions were identified by IPA at the level of *p* < 0.05. The top 5 diseases identified were inflammatory response and disease, neurological disease, immunological disease, organismal injury and abnormalities, and cell death and survival (Supplementary Table 4).

## Discussion

In this pilot study of 5 ICU patients and 5 healthy controls, we postulated and confirmed that patients' 24-h rhythms differ from those of healthy controls. Rhythms were monitored in heart rate, blood pressure, temperature, and circulating plasma metabolites including cortisol. We hypothesized that fewer of these variables would show significant 24-h rhythms in the ICU patient group than in the healthy control group. Our results support this hypothesis. Healthy controls generally had significant 24-h rhythms individually and as a group. On the other hand, while a few ICU patients had significant 24-h rhythms in isolated variables, no significant rhythms were identified in ICU patients at the group level, except in cortisol. This indicates a lack of coherence in rhythm phases and amplitudes among ICU patients. The general lack of a significant rhythm in ICU patients at the population level suggests that 24-h rhythms are synchronized in healthy controls only.

### Sound Levels

Noise levels were significantly louder in ICU patient rooms than in healthy control rooms at all times, and remained elevated at night. In contrast, healthy controls' rooms had a clear decrease in nighttime noise levels.

The World Health Organization recommends that patient care areas not exceed 35 dB LA_eq_ (35). In this study, we observed LA_eq_ levels far above this threshold in ICU patient rooms. Other groups have observed similar trends (15, 37, 38). It has been known for over 20 years that high noise levels are present in the ICU and that they adversely affect sleep (15, 39, 40). There is a clear link between sleep deprivation and delirium, which is related to poor outcomes in ICU patients (11). Because of this link, protocols to improve sleep in the ICU have been well-described (41, 42). However, these interventions do not explicitly control sound levels, nor is it standard clinical practice to control light or sound levels in the ICU. Simple interventions to control ICU light and sound levels and foster sleep are promising, and show a reduction in the incidence of delirium (43).

Though study limitations prevent us from reporting on light levels in study participants' rooms, it is worth commenting on since light is a strong synchronizer of the circadian system. Interestingly, some groups have observed a lack of bright light levels as well as a lack of 24-h variation in light levels in ICU patient rooms, which may contribute to circadian rhythm disruption in patients (16, 17, 44). Protocols to enforce strict light-dark cycles with sufficient brightness in ICU patient rooms during the day should be standard-of-care to help synchronize patient circadian rhythms.

### 24-H Rhythms

Healthy controls had statistically significant or near-significant 24-h rhythms in heart rate, blood pressure, and temperature at both the individual level and the population level. ICU patients did not have significant 24-h rhythms in any of the vitals measured. This finding reflects a disruption to 24-h rhythms in ICU patients, which other groups have noted (6, 45).

We made preliminary identifications of 60 previously identified circadian metabolites in the plasma of study participants using mass spectrometry. Ten of these had a significant 24-h rhythm in the healthy control group. With the exception of cortisol, none of these metabolites showed a significant rhythm in ICU patients at the group level. Even though both ICU patients and healthy controls showed a significant 24-h rhythm in laboratory-quantified cortisol, we noted that patients' cortisol rhythms showed abnormalities in phase and MESOR. Elevated cortisol and disrupted cortisol metabolism are known to occur in critical illness (46, 47). Interestingly, the ICU patient with the lowest APACHE II score has a cortisol pattern that is very close to that of the healthy controls.

It is striking that even in this pilot study, profiled physiological indicators of circadian rhythms showed a significant or near-significant rhythm in healthy controls but not in ICU patients. The data suggest a significant, global disruption to the circadian rhythm in critical illness. The implications of this finding mandate further study.

### Patient Variability

Our work uncovered a surprising clustering pattern in plasma metabolic profiles. Samples from ICU patients formed very distinct clusters corresponding to each individual patient, while samples from healthy controls clustered tightly together, with no resolution at the level of the individuals providing the samples. The ICU patient with plasma samples clustered nearest to the group of healthy controls (red dots in Figure 4) had a similar cortisol rhythm, a lower APACHE II score, and a shorter ICU stay than the other patients. Therefore, we hypothesize that the patient-level resolution of the metabolomics data shown in Figure 4 is linked to an individual patient's state, and that multiple-day sampling could show patient “trajectories” that move toward improved or worsening health. However, we could discern no other pattern in the PCA scores in this small study. While other researchers have associated metabolomics data with mortality in critical illness (48), our finding raises the possibility that metabolomics may provide broad insight into individual patients' conditions. More focused research will have to be done to confirm and explain the highly individualized patterns in sample clustering observed in ICU patients.

### Disruptions to Metabolism

Restricting the untargeted metabolomics data from 25,000 features to those that differ between ICU patients and healthy controls at the level of *q* < 0.05 only reduced the data set to 15,000 features. These results indicate that ICU patients have highly disordered metabolism relative to healthy controls.

To explore this, we analyzed the untargeted metabolomics data with Ingenuity Pathway Analysis. Interestingly, each of the top 5 dysregulated pathways identified by IPA have been linked to the circadian system. Eicosanoids are broadly involved in inflammation, vasoconstriction, pain perception, and cell growth and regulation. Prostanoids, a class of eicosanoids, have long been known to oscillate with a daily rhythm in saliva (49). The FXR/RXR activation pathway modulates bile acid, lipid, and glucose metabolism. Bile acid synthesis, FXR, and RXR are all linked by the circadian system (50, 51). Finally, spermines and spermidines belong to the polyamide class of molecules and regulate various genetic processes. Polyamine biosynthesis has also been shown to intersect with the circadian system (52).

This pilot study had several limitations. We did not use a circadian protocol with healthy controls or ICU patients. As such, we are unable to clearly state whether the observed differences in 24-h rhythms are due to endogenous factors, exogenous factors, or both. We use the term “24-h rhythms” instead of “circadian rhythms” because of this. Only 5 individuals per group were studied. Future work must include larger, more uniform patient groups. All 5 ICU patients had different underlying conditions. A more focused study of patient groups with distinct underlying conditions (e.g., sepsis vs. stroke) should be done to evaluate how individual diseases contribute to circadian rhythm desynchronization and to observed patterns in the metabolomics data. Core body temperature should be measured in all participants. The lack of significant rhythm in temperature observed in healthy controls (*p* = 0.091) may be due to this.

Only positive mode reverse phase MS1 data were collected. Future work should take a targeted approach such as the Biocrates Absolute IDQ kits used in similar research (26). The metabolite identification reported here is preliminary and should be confirmed in future work. Melatonin was not conclusively identified in our mass spectrometry data and should be quantified by mass spectrometry and/or radio immunoassay in future work. The IPA results should be considered preliminary as well.

More frequent sampling of blood for metabolomics data would improve profiling of circadian rhythms (53). The lack of statistical significance in individual rhythms of metabolites may be alleviated by more data. Plasma samples could be collected at a higher frequency, or for a longer period of time, or both. This supports our case that a larger study is needed.

Finally, we recorded bedside light levels for all study participants. However, our light meter had to be replaced halfway through the study and we did not feel that the data from the two separate meters could be meaningfully compared. We have opted to not present these data in the manuscript. Despite these limitations, the data clearly show a global disruption to 24-h rhythms in ICU patients as well as fascinating individual patterns in ICU patients' metabolomes. Both results deserve further study.

## Conclusions

This pilot study confirms the literature supporting a broad desynchronization of circadian rhythms in ICU patients on multiple levels by measuring circadian rhythms in vital signs and plasma metabolites. In addition, PCA of untargeted metabolomics identifies a strong pattern in metabolomes of ICU patients, which are highly individualized and distinct from healthy controls. These results suggest a significant disordering of physiology in ICU patients, involving at a minimum the metabolism and the circadian system, which should be characterized further in order to determine the implications for patient care.

## Data Availability Statement

The datasets generated for this study can be found in the https://doi.org/10.13020/7TFS-SF98.

## Ethics Statement

The studies involving human participants were reviewed and approved by University of Minnesota Institutional Review Board. The patients/participants provided their written informed consent to participate in this study.

## Author Contributions

EL was study PI. She contributed to study design, data analysis and interpretation, and prepared the manuscript. JE and LP performed data analysis, assisted with manuscript preparation, and approved the final manuscript. SH obtained mass spectrometry data, contributed to manuscript preparation, and approved the final manuscript. MS and SM identified patients, collected data, and approved the final manuscript. GB contributed to study design and data interpretation and approved the final manuscript. GC-G contributed to study design, data analysis and interpretation, manuscript preparation, and approved the final manuscript. All authors contributed to the article and approved the submitted version.

## Conflict of Interest

The authors declare that the research was conducted in the absence of any commercial or financial relationships that could be construed as a potential conflict of interest.
