# Supplementary material for: Metabolomics Pilot Study Identifies Desynchronization of 24-H Rhythms and Distinct Intra-patient Variability Patterns in Critical Illness: A Preliminary Report
Source: Front Neurol. 2020 Oct 2;11:533915. doi: 10.3389/fneur.2020.533915 (PMC7566909; doi:10.3389/fneur.2020.533915)
Supplement: Supplementary file 1 [file Data_Sheet_1.zip › Suppl Data Sheet 1 - IndividualPlots.pdf]

Time series plots of vitals and circadian metabolites putatively identified with mass spectrometry. Healthy controls are shown in black and ICU patients are shown in red.

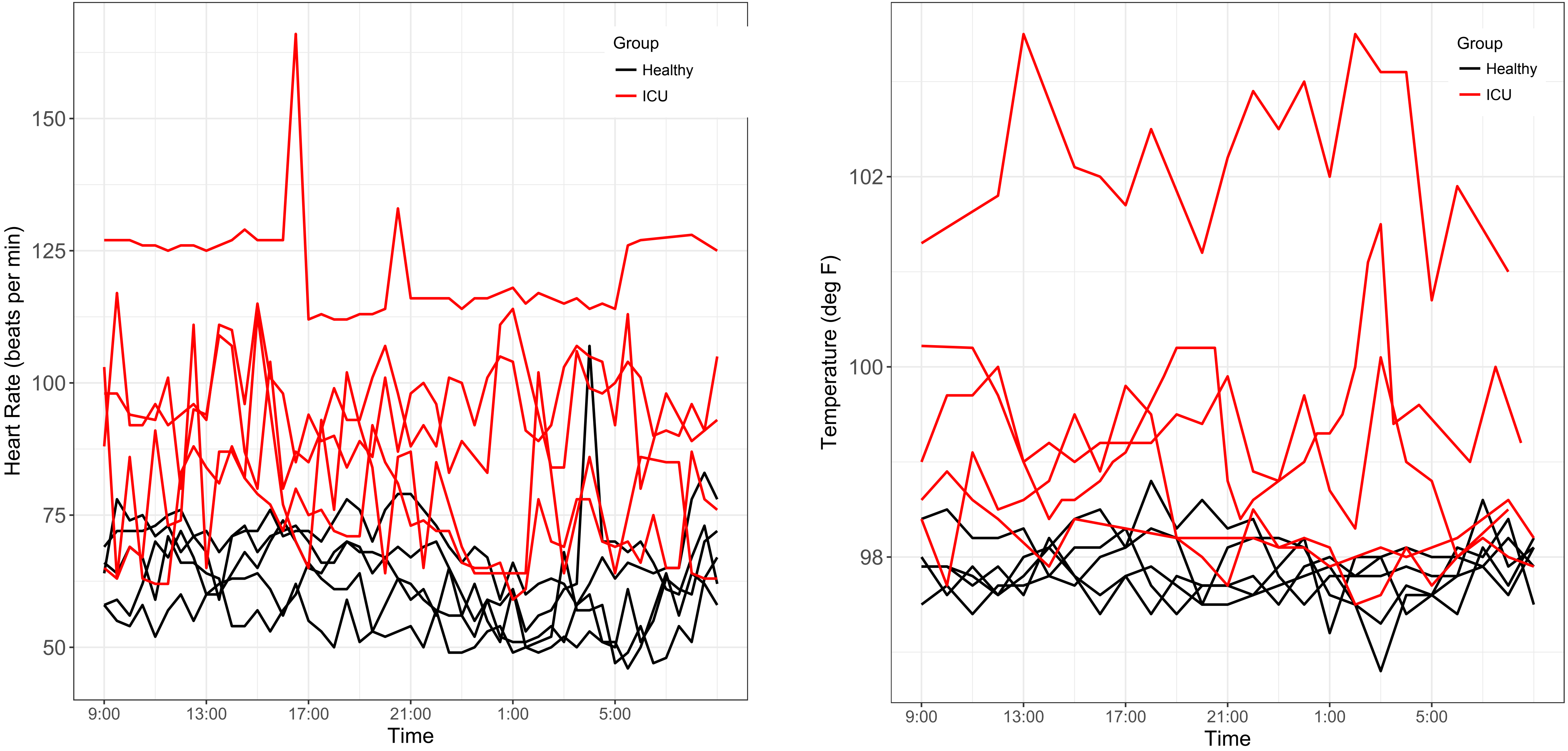

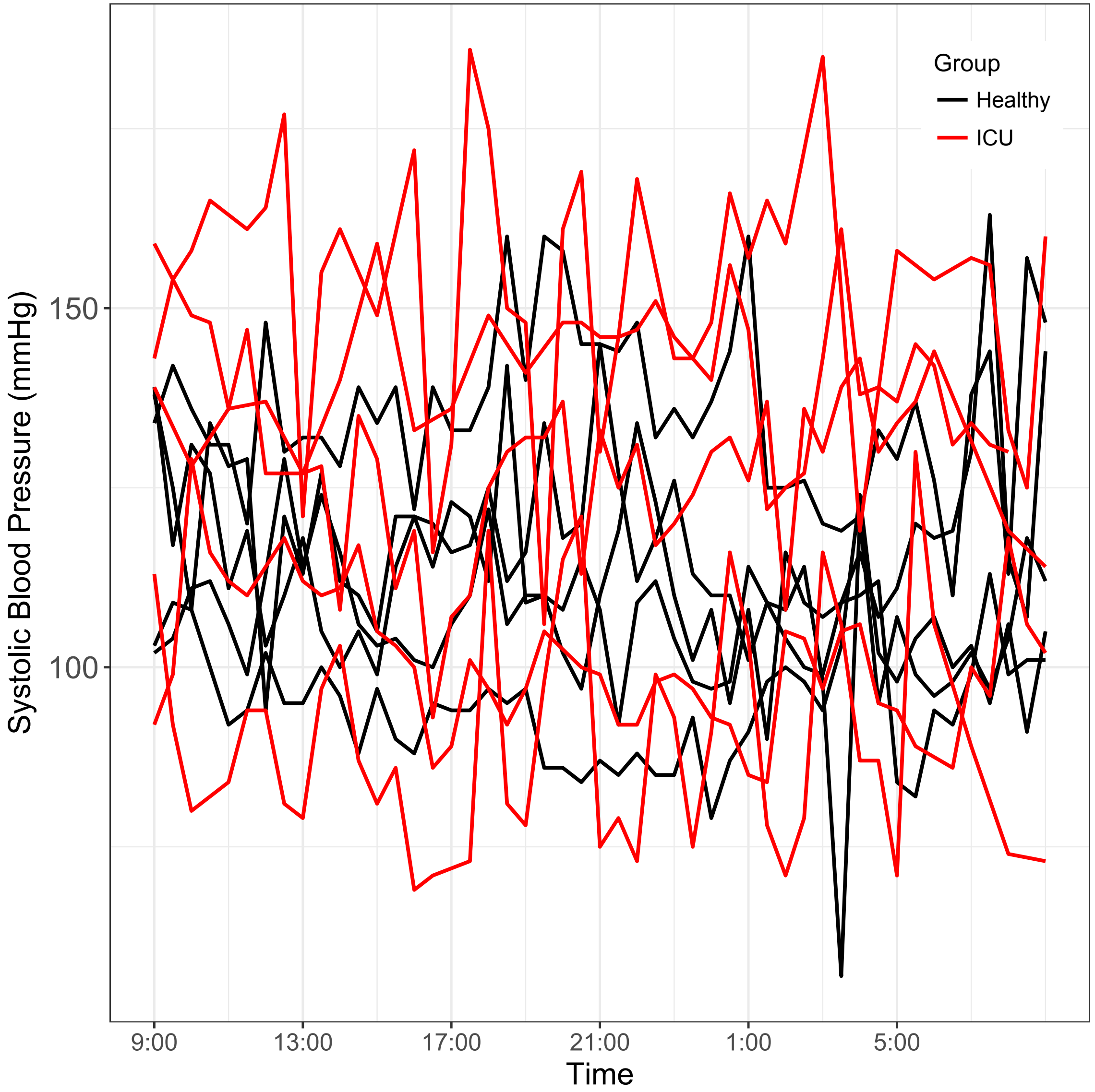

Systolic Blood Pressure (mmHg)
150
100
9:00
13:00
17:00
21:00
1:00
5:00
Time
Group
Healthy
ICU

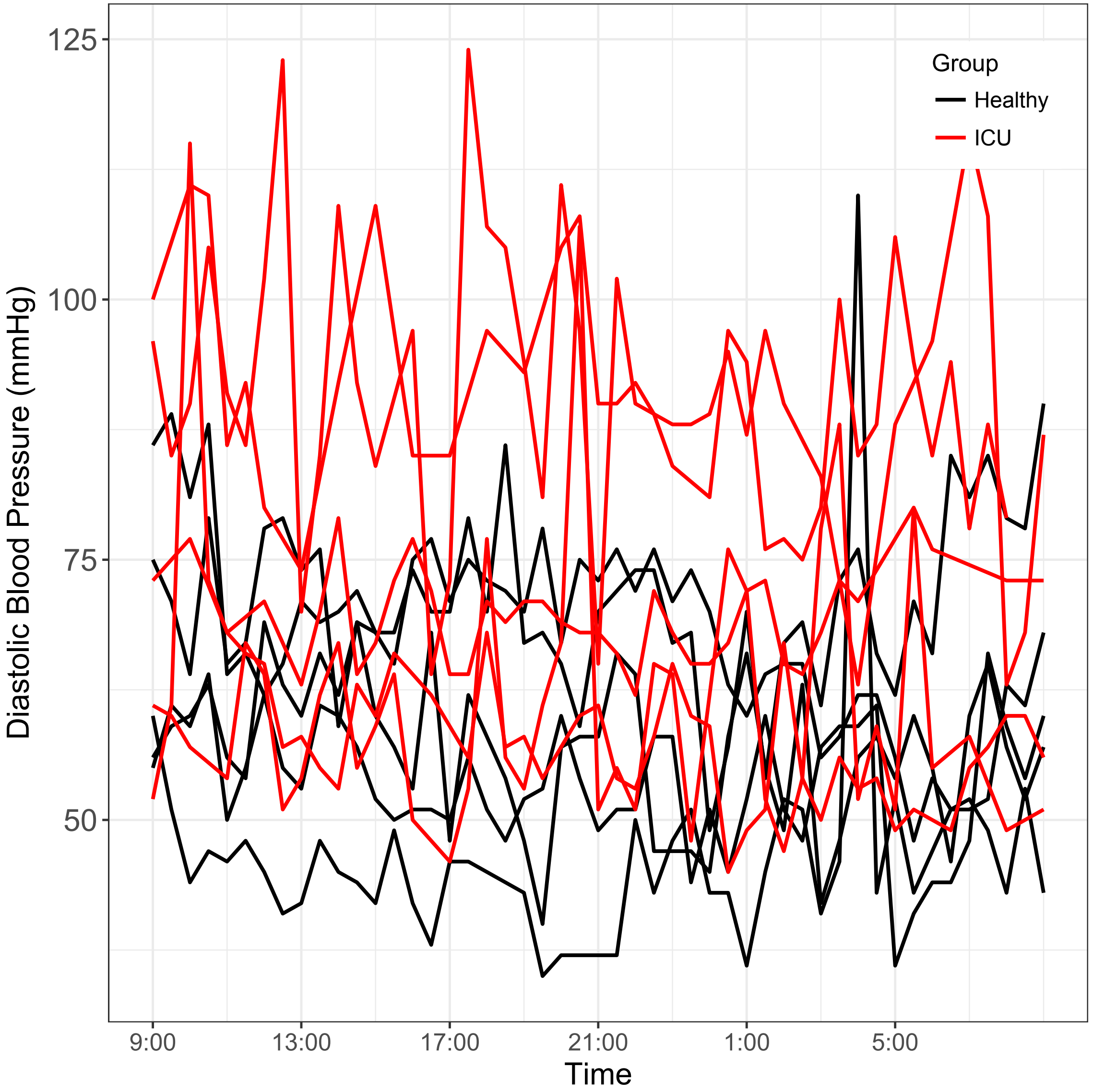

Diastolic Blood Pressure (mmHg)
125
100
75
50
9:00
13:00
17:00
21:00
1:00
5:00
Time
Group
Healthy
ICU

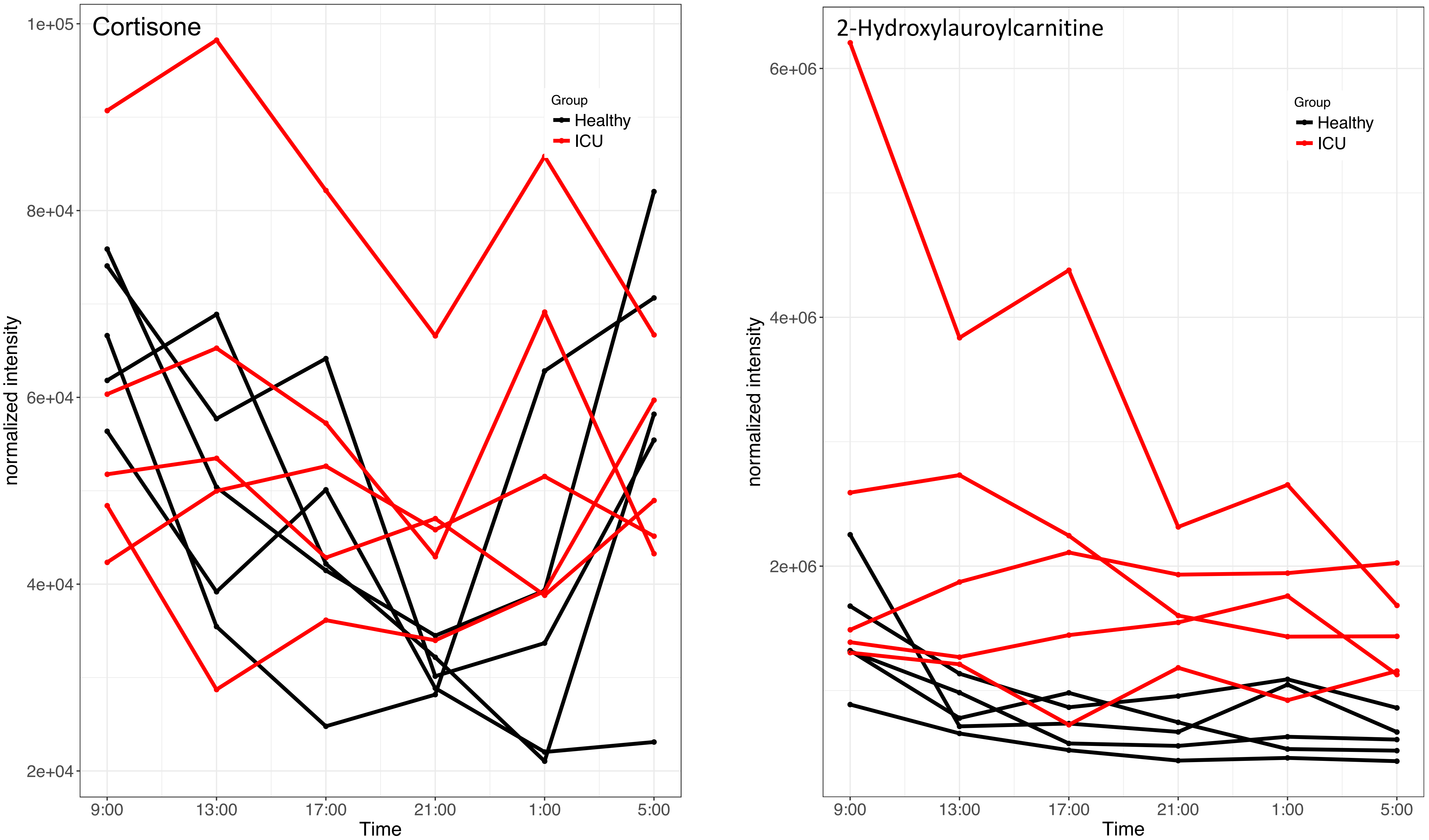
Cortisone
1e+05
8e+04
6e+04
4e+04
2e+04
normalized intensity
Group
Healthy
ICU
9:00
13:00
17:00
21:00
1:00
5:00
Time
2-Hydroxylauroylcarnitine
6e+06
4e+06
2e+06
normalized intensity
Group
Healthy
ICU
9:00
13:00
17:00
21:00
1:00
5:00
Time

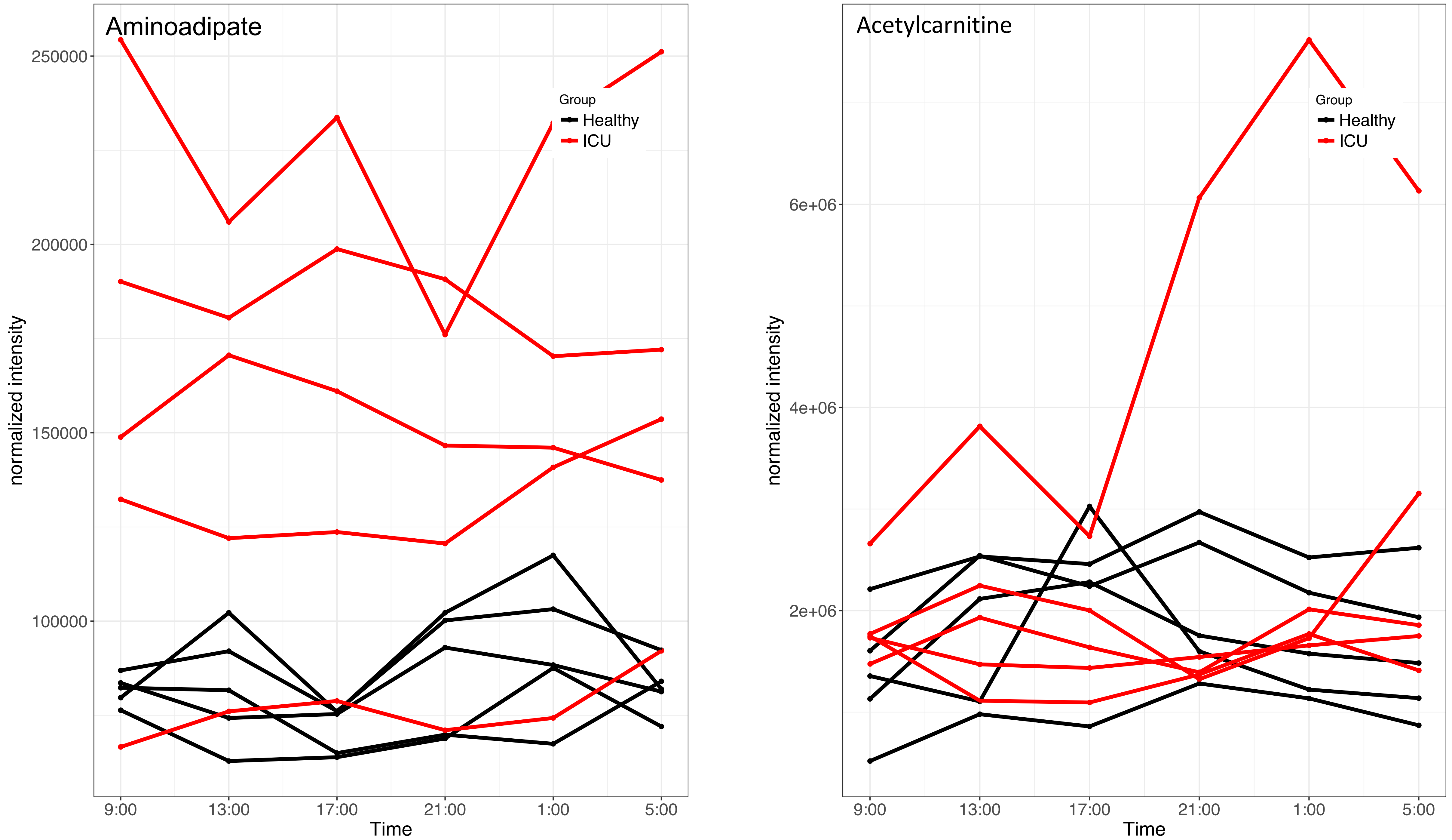
Aminoadipate
250000
200000
150000
100000
normalized intensity
Group
Healthy
ICU
9:00
13:00
17:00
21:00
1:00
5:00
Time
Acetylcarnitine
6e+06
4e+06
2e+06
normalized intensity
Group
Healthy
ICU
9:00
13:00
17:00
21:00
1:00
5:00
Time

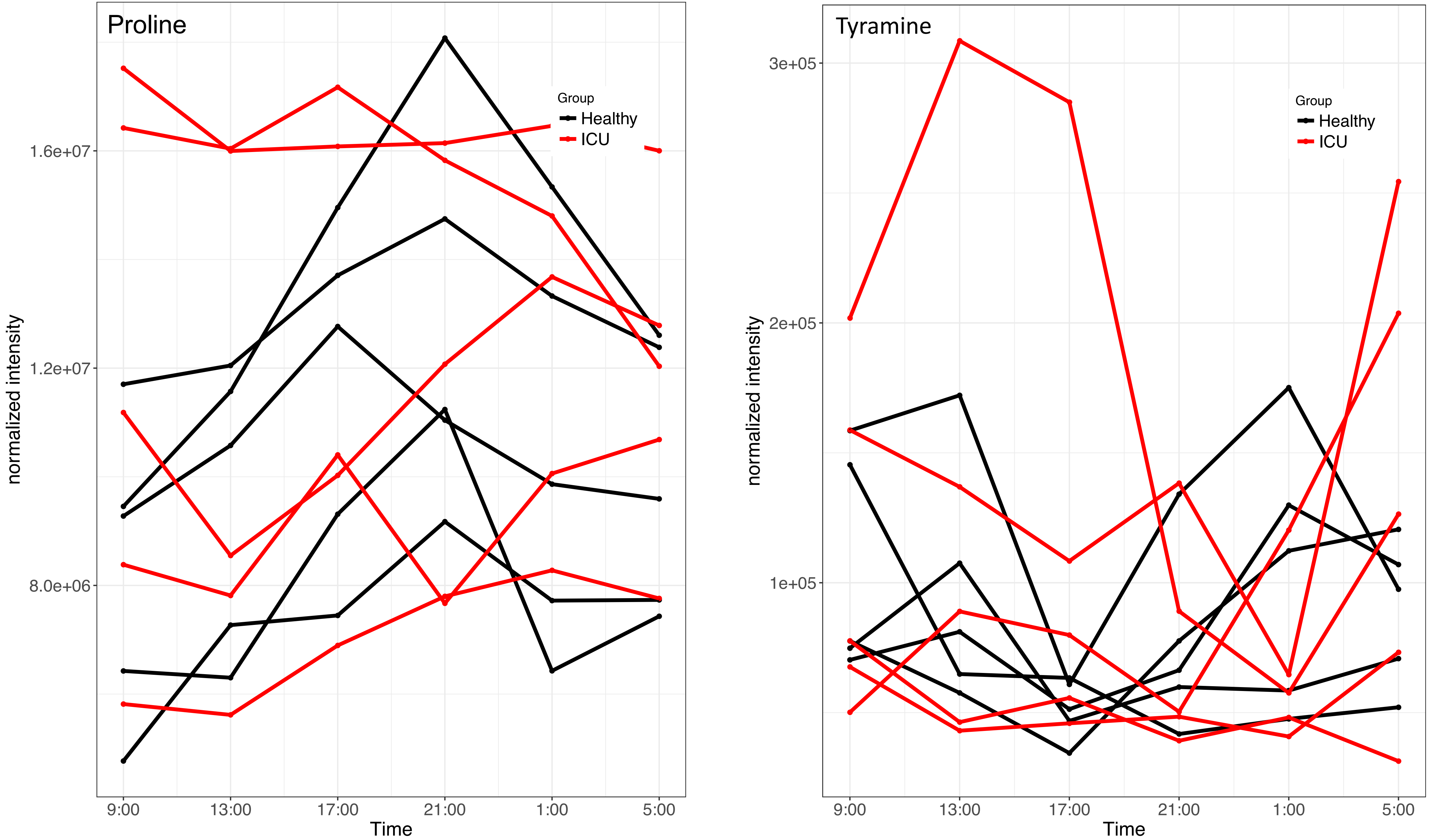

Proline
Group
Healthy
ICU
1.6e+07
1.2e+07
8.0e+06
normalized intensity
9:00
13:00
17:00
21:00
1:00
5:00
Time
Tyramine
Group
Healthy
ICU
3e+05
2e+05
1e+05
normalized intensity
9:00
13:00
17:00
21:00
1:00
5:00
Time

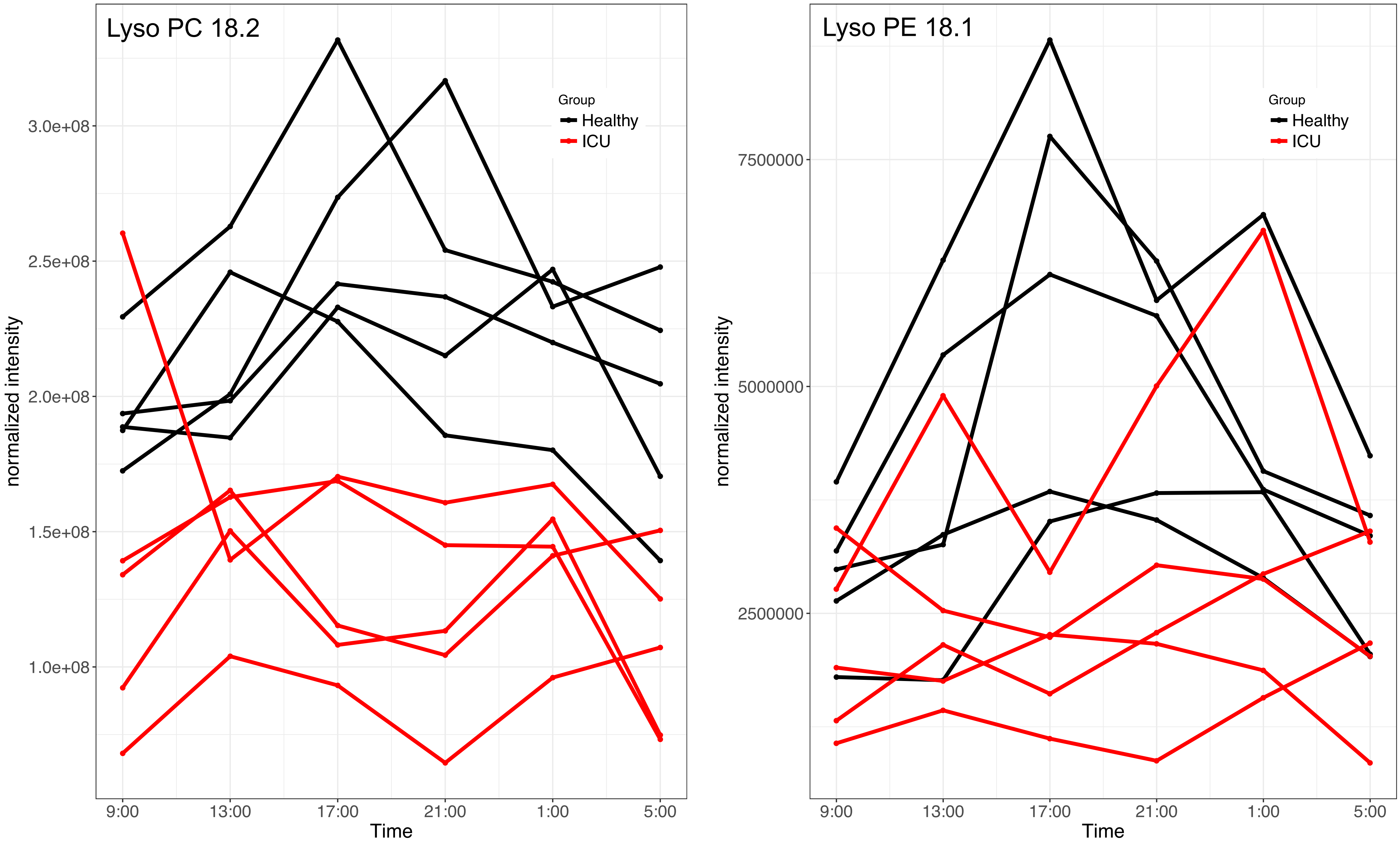
Lyso PC 18.2
Group
Healthy
ICU
3.0e+08
2.5e+08
2.0e+08
1.5e+08
1.0e+08
normalized intensity
9:00
13:00
17:00
21:00
1:00
5:00
Time
Lyso PE 18.1
Group
Healthy
ICU
7500000
5000000
2500000
normalized intensity
9:00
13:00
17:00
21:00
1:00
5:00
Time

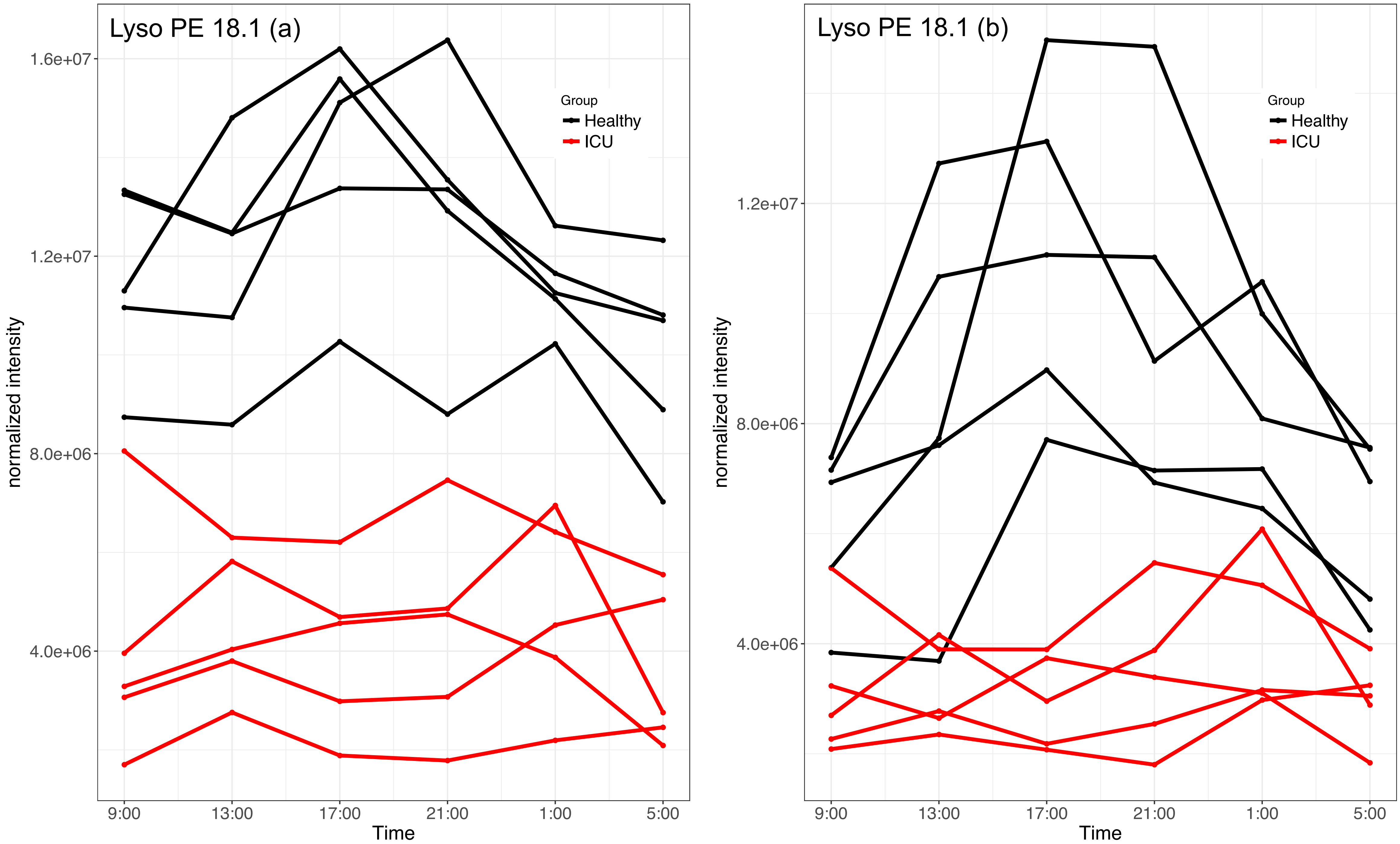

Lyso PE 18.1 (a)
1.6e+07
1.2e+07
8.0e+06
4.0e+06
normalized intensity
9:00
13:00
17:00
21:00
1:00
5:00
Time
Group
Healthy
ICU
Lyso PE 18.1 (b)
1.2e+07
8.0e+06
4.0e+06
normalized intensity
9:00
13:00
17:00
21:00
1:00
5:00
Time
Group
Healthy
ICU
