# Supplementary material for: Metabolomics Pilot Study Identifies Desynchronization of 24-H Rhythms and Distinct Intra-patient Variability Patterns in Critical Illness: A Preliminary Report
Source: Front Neurol. 2020 Oct 2;11:533915. doi: 10.3389/fneur.2020.533915 (PMC7566909; doi:10.3389/fneur.2020.533915)
Supplement: Supplementary file 1 [file Data_Sheet_1.zip › Suppl Data Sheet 2 - MetabolitePlot.pdf]

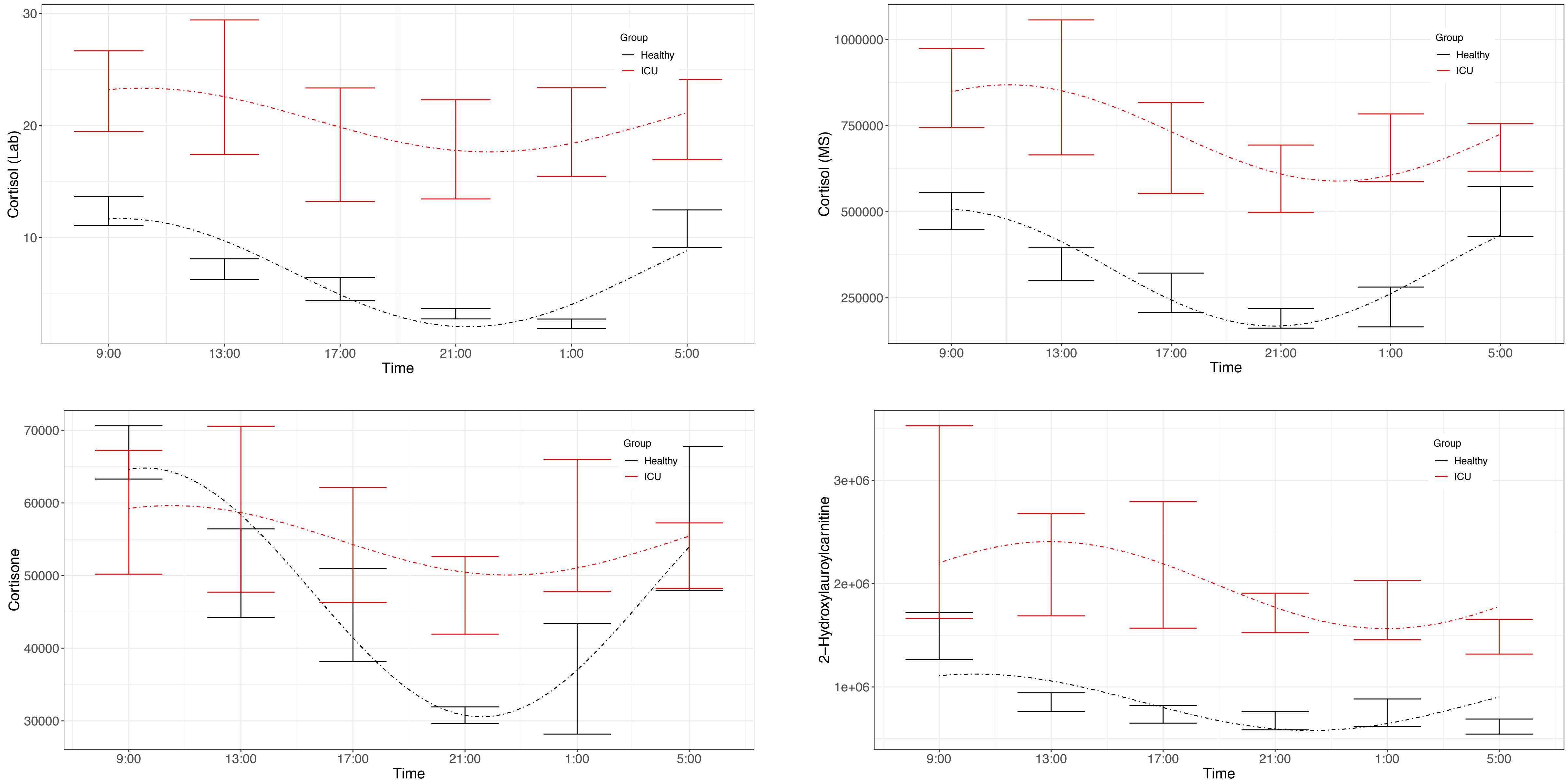

Time series plots of circadian metabolites putatively identified with mass spectrometry. Spectral intensities are plotted against time as mean with standard error bars. Cosinor fits of the 24-hour rhythm are shown in dotted lines. Red indicates ICU patients and black indicates healthy controls. Two features were ambiguously assigned the identity Lyso PE 18.2 and both are shown.

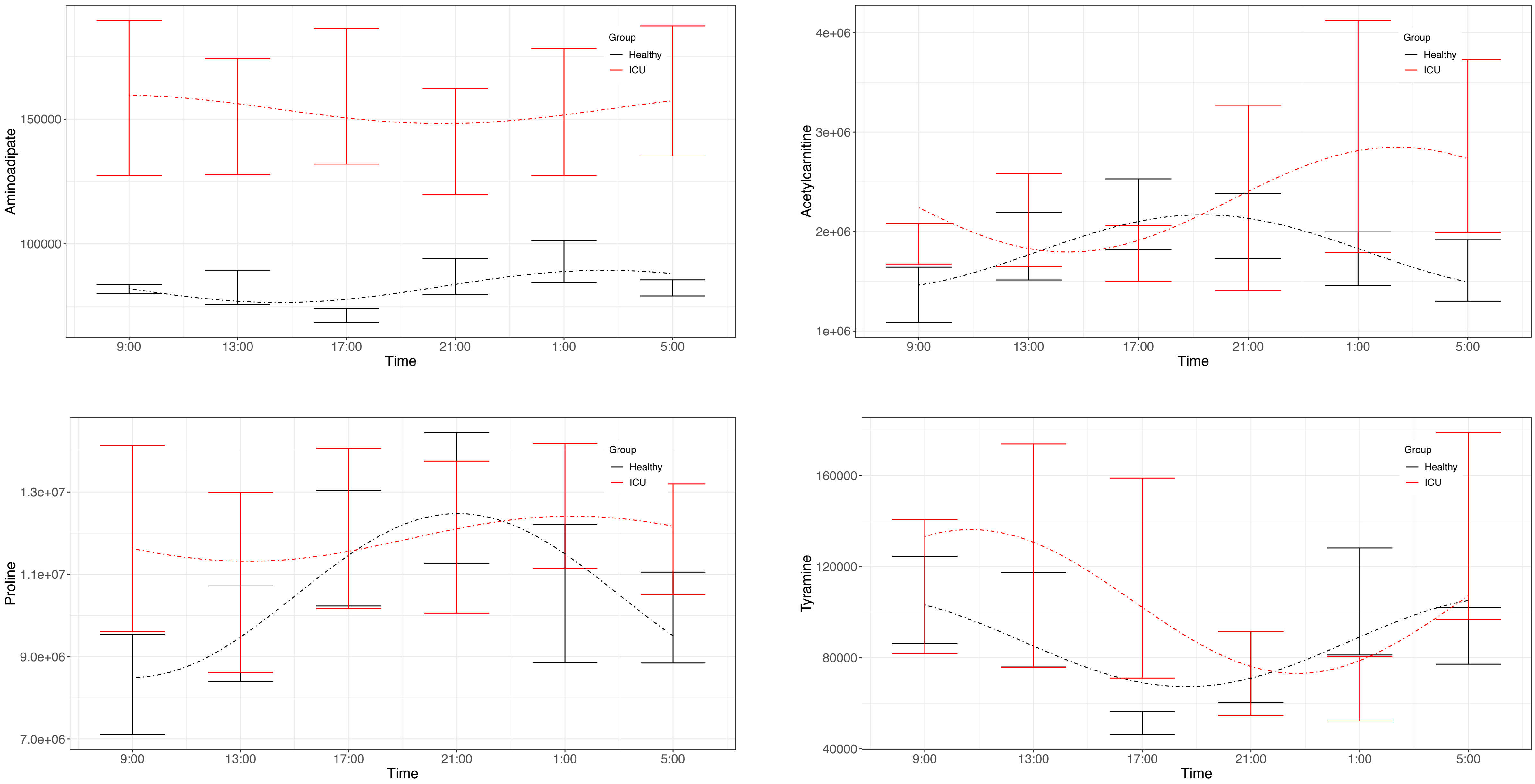

Time series plots of circadian metabolites putatively identified with mass spectrometry. Spectral intensities are plotted against time as mean with standard error bars. Cosinor fits of the 24-hour rhythm are shown in dotted lines. Red indicates ICU patients and black indicates healthy controls. Two features were ambiguously assigned the identity Lyso PE 18.2 and both are shown.

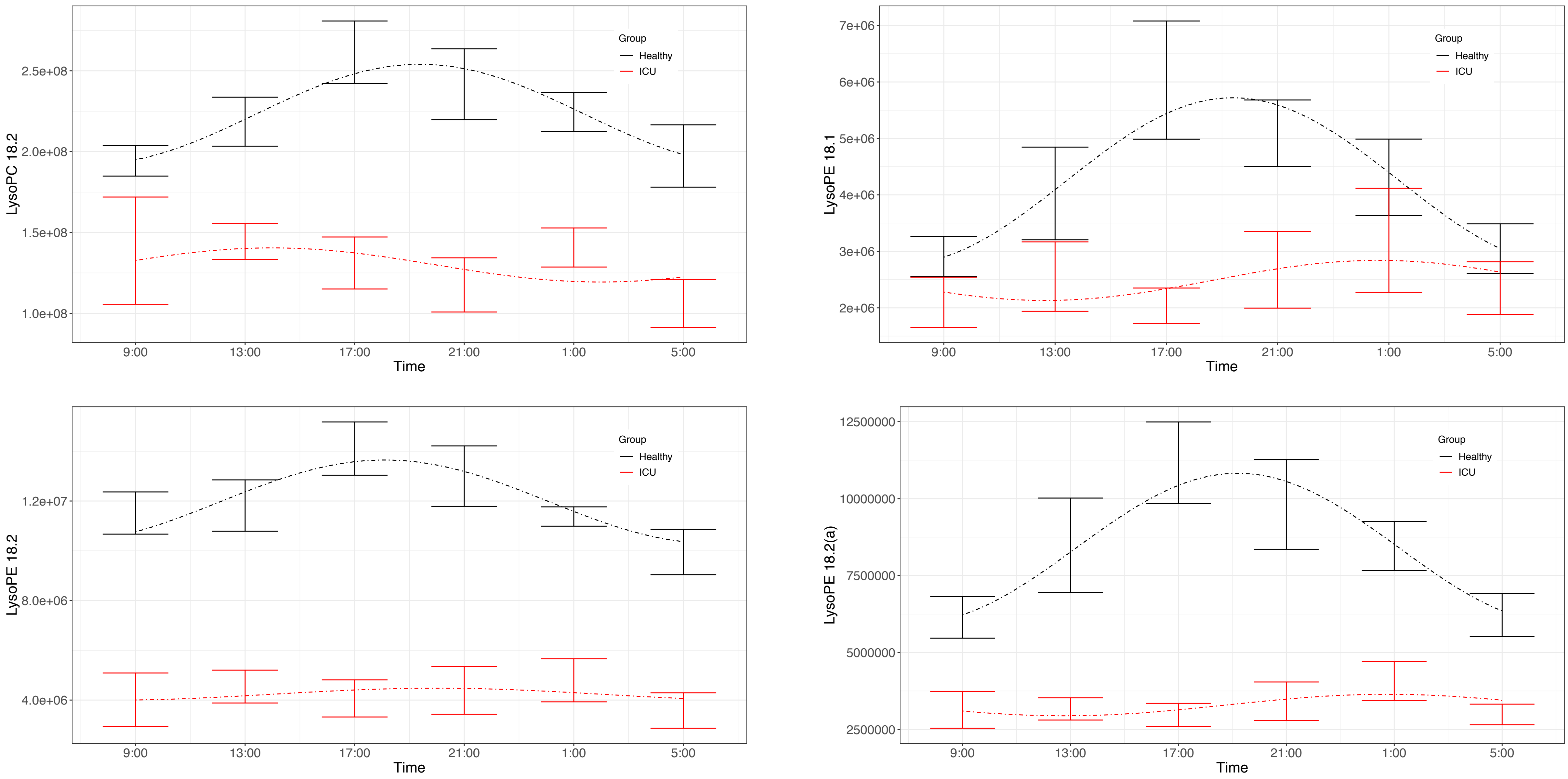

Time series plots of circadian metabolites putatively identified with mass spectrometry. Spectral intensities are plotted against time as mean with standard error bars. Cosinor fits of the 24-hour rhythm are shown in dotted lines. Red indicates ICU patients and black indicates healthy controls. Two features were ambiguously assigned the identity Lyso PE 18.2 and both are shown.
