# Supplementary material for: Metabolomics Pilot Study Identifies Desynchronization of 24-H Rhythms and Distinct Intra-patient Variability Patterns in Critical Illness: A Preliminary Report
Source: Front Neurol. 2020 Oct 2;11:533915. doi: 10.3389/fneur.2020.533915 (PMC7566909; doi:10.3389/fneur.2020.533915)
Supplement: Supplementary file 1 [file Data_Sheet_1.zip › Suppl Figure 1 - Cortisol.pdf]

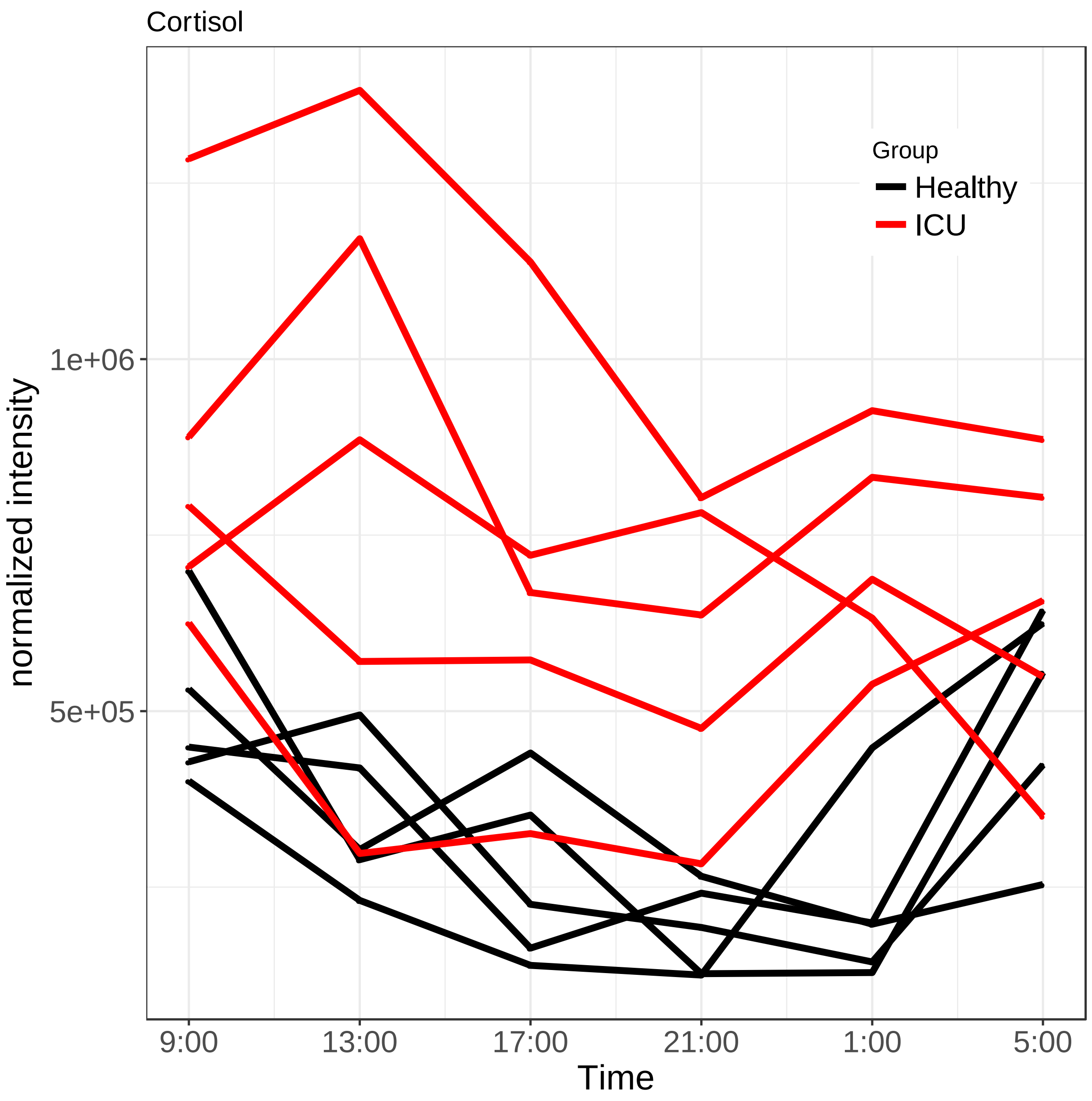

Supplementary Figure 5: Cortisol spectral intensities quantified with mass spectrometry. Overall there is good agreement with the laboratory-quantified cortisol concentrations.
