# Supplementary material for: Metabolomics Pilot Study Identifies Desynchronization of 24-H Rhythms and Distinct Intra-patient Variability Patterns in Critical Illness: A Preliminary Report
Source: Front Neurol. 2020 Oct 2;11:533915. doi: 10.3389/fneur.2020.533915 (PMC7566909; doi:10.3389/fneur.2020.533915)
Supplement: Supplementary file 1 [file Data_Sheet_1.zip › Suppl Table 1 Standards Table.pdf]

Supplemental Table 1: Deuterated internal standards

Compound	MW	+H	-H	Concentration
Phenylalanine-D5	170.10981	171.11764	169.10179	50ug/mL
Hippuric Acid-D5	184.08908	185.0969	183.08111	50ug/mL
Cholic acid-D4	412.31213	413.31996	411.3054	50ug/mL
Glucose-D7		187.10678	188.1146	186.10005
		210.09655(Na)	222.0762 (Cl)	
C16:0-D3 (palmitic acid)			258.25138	20ug/mL

Deuterated internal standards were obtained from CDN Isotopes and Cambridge isotopes Laboratories. MW = molecular weight
