# Supplementary material for: Metabolomics Pilot Study Identifies Desynchronization of 24-H Rhythms and Distinct Intra-patient Variability Patterns in Critical Illness: A Preliminary Report
Source: Front Neurol. 2020 Oct 2;11:533915. doi: 10.3389/fneur.2020.533915 (PMC7566909; doi:10.3389/fneur.2020.533915)
Supplement: Supplementary file 1 [file Data_Sheet_1.zip › Suppl Table 2 - BMI Table.pdf]

Supplemental Table 2: BMI Data

**Patient ID (Healthy)**	**BMI**	**Patient ID (ICU)**	**BMI**
P01	31.6	P07	31.0
P02	22.5	P08	37.1
P03	22.4	P10	35.1
P05	30.3	P11	37.2
P06	33.3	P12	57.3

BMI data for healthy controls and ICU patients. At the group level, BMI did not significantly differ between healthy controls and ICU patients ($p=0.07$).
